# The conserved upstream region of *lscB/C* determines expression of different levansucrase genes in plant pathogen *Pseudomonas syringae*

**DOI:** 10.1186/1471-2180-14-79

**Published:** 2014-03-27

**Authors:** Shaunak Khandekar, Abhishek Srivastava, Daniel Pletzer, Antje Stahl, Matthias S Ullrich

**Affiliations:** 1 Molecular Life Sciences Research Center, Jacobs University Bremen, Campus Ring 1, Bremen, 28759, Germany; 2 Current Address: Department of Experimental Limnology, Leibniz-Institute of Freshwater Ecology and Inland Fisheries, Alte Fischerhuette 2, Stechlin, 16775, Germany

**Keywords:** *Pseudomonas syringae*, Levansucrase, Expression, Exopolysaccharides, Levan, Evolution

## Abstract

**Background:**

*Pseudomonas syringae* pv. glycinea PG4180 is an opportunistic plant pathogen which causes bacterial blight of soybean plants. It produces the exopolysaccharide levan by the enzyme levansucrase. Levansucrase has three gene copies in PG4180, two of which, *lscB* and *lscC*, are expressed while the third, *lscA*, is cryptic. Previously, nucleotide sequence alignments of *lscB/C* variants in various *P. syringae* showed that a ~450-bp phage-associated promoter element (PAPE) including the first 48 nucleotides of the ORF is absent in *lscA*.

**Results:**

Herein, we tested whether this upstream region is responsible for the expression of *lscB/C* and *lscA.* Initially, the transcriptional start site for *lscB/C* was determined. A fusion of the PAPE with the ORF of *lscA* (*lscB*_*UpN*_*A*) was generated and introduced to a levan-negative mutant of PG4180. Additionally, fusions comprising of the non-coding part of the upstream region of *lscB* with *lscA* (*lscB*_*Up*_*A*) or the upstream region of *lscA* with *lscB* (*lscA*_*Up*_*B*) were generated. Transformants harboring the *lscB*_*UpN*_*A* or the *lscB*_*Up*_*A* fusion, respectively, showed levan formation while the transformant carrying *lscA*_*Up*_*B* did not. qRT-PCR and Western blot analyses showed that *lscB*_*UpN*_*A* had an expression similar to *lscB* while *lscB*_*Up*_*A* had a lower expression. Accuracy of protein fusions was confirmed by MALDI-TOF peptide fingerprinting.

**Conclusions:**

Our data suggested that the upstream sequence of *lscB* is essential for expression of levansucrase while the N-terminus of LscB mediates an enhanced expression. In contrast, the upstream region of *lscA* does not lead to expression of *lscB*. We propose that *lscA* might be an ancestral levansucrase variant upstream of which the PAPE got inserted by potentially phage-mediated transposition events leading to expression of levansucrase in *P. syringae*.

## Background

*Pseudomonas syringae* comprises a large and well-studied group of plant-pathogenic bacteria [1]. They infect a broad range of host plants and are subdivided into more than 50 different pathogenic variants called pathovars [2]. *P. syringae* possesses a number of well-studied virulence and pathogenicity factors such as the Type III effector trafficking system, various phytotoxins, different mechanisms suppressing the plant defense, or synthesis of exopolysaccharides [3-5]. Exopolysaccharides play a variety of roles in virulence and pathogenicity not only in *Pseudomonas* but also in other biofilm-producing organisms [6,7]. The two major exopolysaccharides produced by *P. syringae* pv. glycinea are alginate and levan [7]. Levan is a β-(2,6) polyfructan with extensive branching through β-(2,1) linkages, while alginate is a copolymer of O-acetylated β-(1,4)-linked D-mannuronic acid and its C-5 epimer, L-guluronic acid [7-10].

*P. syringae* pv. glycinea PG4180 causes bacterial blight of soybean plants. Like some other *Pseudomonas* species, this organism utilizes sucrose as a carbon source with the help of the enzyme levansucrase (EC 2.4.1.10, Lsc), in the process releasing glucose and forming the exopolysaccharide levan. PG4180 produces no alginate due to a native frameshift mutation in the *algT* gene and hence, the exopolysaccharide matrix of this strain is mainly composed of levan [11]. Additionally to several draft genome sequences [12-18], the complete genome sequences of three *P. syringae* pathovars are available, namely pv. tomato DC3000 [19], pv. phaseolicola 1448A [20] and pv. syringae B728a [21]. These strains serve as excellent model organisms to study plant-microbe interactions. Like in some other *P. syringae* pathovars, the PG4180 genome contains three copies of the *lsc* gene, of which two – *lscA* and *lscC* – are chromosomally encoded while *lscB* is plasmid-encoded. Of the three copies, only *lscB* and *lscC* have been shown to be expressed while no expression was observed for *lscA* under the tested growth conditions since a mutant, PG4180.M6, lacking *lscB* and lscC but containing *lscA* was levan-negative [10]*.* Interestingly, the ORF coding for LscA is fully functional since this gene from pv. glycinea, and its homologues from pv. phaseolicola and pv. tomato, could be expressed from recombinant promoters in *Escherichia coli*[9,22]. Even though LscB is predominantly extra-cellular and LscC is predominantly retained in the periplasm, the two enzymes are 98% identical at the amino acid level [23]. There are only five amino acid residues different, four of which are conserved changes. Since the enzymes are highly similar in their structure as well as function, all experiments in this study were done using *lscB* only.

As reported by Srivastava *et al.*[24], nucleotide sequence comparison of the *lscA* variants with those of *lscB/C* variants of *P. syringae* pathovars showed that the first 48-bp of the N-terminus of the ORF *lscB/C* were absent in *lscA. In silico* removal of this N-terminal region increased the identity from 87.5% to 93% at the amino acid residue sequence level between LscA and B/C variants. The comparison also showed that a ~450-bp upstream region, which is highly conserved in all *lscB*/*C* variant loci, is missing upstream of *lscA*. This region spanning from −450-bp to +48-bp with respect to the translational start site of *lscB/C* was predicted to be a pro-phage borne DNA based on sequence similarities and hence was termed phage-associated promoter element (PAPE) [24].

*P. syringae* is the only Lsc-synthesizing organism having multiple gene copies coding for this enzyme. The rationale for the occurrence of multiple *lsc* gene copies, some of which carry upstream PAPEs, remained obscure and prompted the current study, during which the transcriptional start site of *lscB/C* was determined to be -339 bp upstream to the translational start codon. Subsequently, the PAPE with or without the N-terminal coding sequence was fused to *lscA*. Additionally, the upstream region of *lscA* was fused with the coding sequence of *lscB* while *lscB* and *lscA* with their native upstream sequences served as controls. All fusion constructs were expressed in the levan-negative mutant PG4180.M6 [10], and tested for their levan formation ability by zymographic detection followed by matrix-assisted laser desorption/ionization time of flight (MALDI-TOF) analysis as well as by Western blotting. Furthermore, the expression of the fusions at the mRNA level was checked by qRT-PCR analysis. In addition, a PCR approach with cDNA was undertaken to show that the expression of *lscA* is also cryptic in other *P. syringae* pathovars.

## Results

### Determination of the transcriptional start site of *lscB*

**Figure 1:**
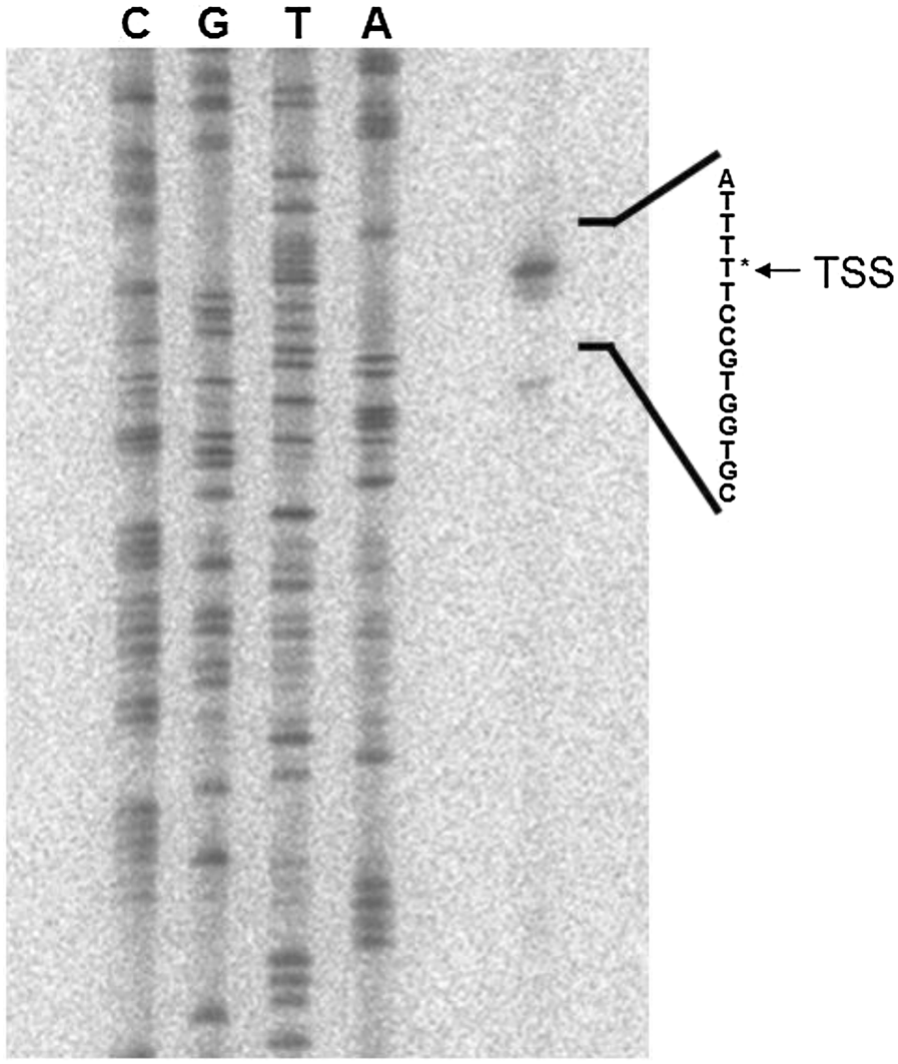
**Determination of the transcriptional start site (TSS) of *lscB *in *P. syringae *pv. glycinea PG4180.** The TSS was determined by electrophoresis of nucleotide sequencing reaction and primer extension product using primer pe.BC.PG ~ 150 bp on 6% polyacrylamide gel. Nucleotide of the TSS (*) is shown at the right.

The coding regions and upstream sequences of *lscB*/C are highly identical to each other (98.1% DNA identity for the coding sequences and 97.5% DNA identity for the 500-bp upstream sequences). As shown by Srivastava *et al*., a deletion construct ending at position −332-bp with respect to the *lscB* translational start codon does not lead to levan formation in levan negative mutant PG4180.M6 while the construct ending −440-bp leads to levan formation in the same mutant [24]. Consequently, primer extension experiments using total RNA from PG4180 cells and a set of reverse oligonucleotide primers were used to determine the transcriptional start site (TSS) of the *lscB* gene. Resolving the extension products on a polyacrylamide gel resulted in a clear signal at nucleotide position −339-bp upstream of the translational start codon of *lscB* (Figure 1). The experiments were repeated for *lscC* giving identical results (Data not shown).

### Qualitative analysis of *lsc* fusion proteins

**Figure 2:**
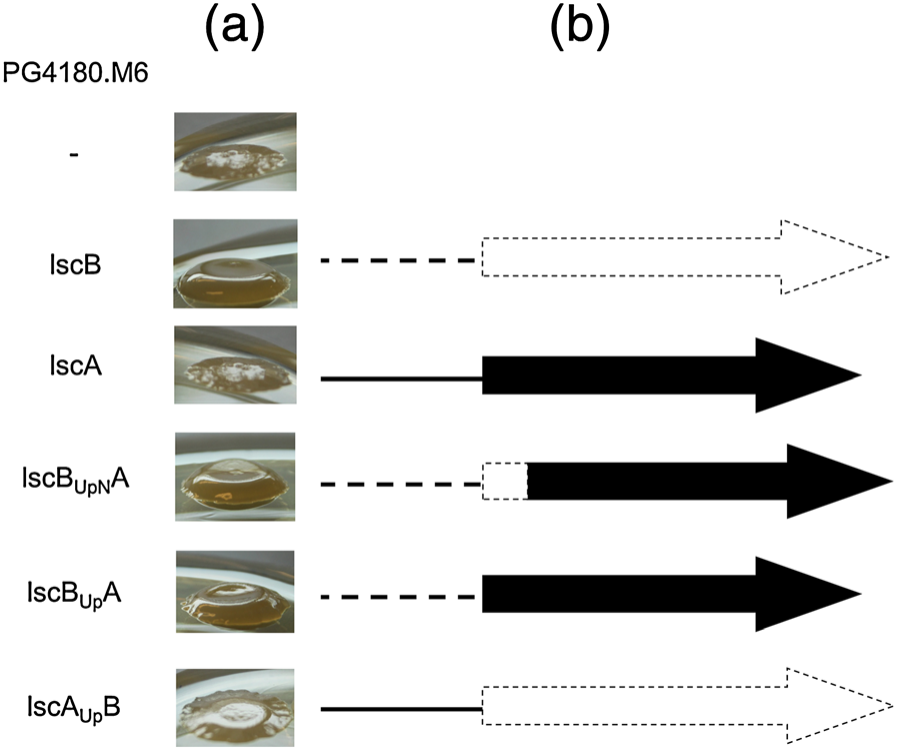
**Illustration of the different *lsc *genes and fusion constructs. (a)** Levan formation ability of the proteins encoded by the fusion constructs in levan negative mutant PG4180.M6. The cells were grown on mannitol-glutamate agar medium containing 5% sucrose at 18°C to check for levan formation (indicated by the dome-shaped glossy slime) around the colony. LscB, LscB_UpN_A and LscB_Up_A showed levan formation. **(b)** Schematic representation of the DNA fusion products. The dashed line and dashed arrow represents *lscB* while the solid line and solid arrow represents *lscA*.

The fusion constructs were introduced to the levan-negative mutant PG4180.M6 and were first analyzed for their levan forming ability on sucrose supplemented mannitol-glutamate agar plates. Both, the PG4180.M6 mutant complemented with *lscB*_*UpN*_*A* and *lscB*_*Up*_*A*, showed levan formation indistinguishable from that of the PG4180.M6 mutant complemented with *lscB* (Figure 2). In contrast, PG4180.M6 complemented with *lscA*_*Up*_*B* was levan negative, same as PG4180.M6 transformed with *lscA*, thus, suggesting that the upstream region of *lscB* mediates expression of downstream located genes while that of *lscA* does not.

### Characterization of *lsc* fusion proteins

**Figure 3:**
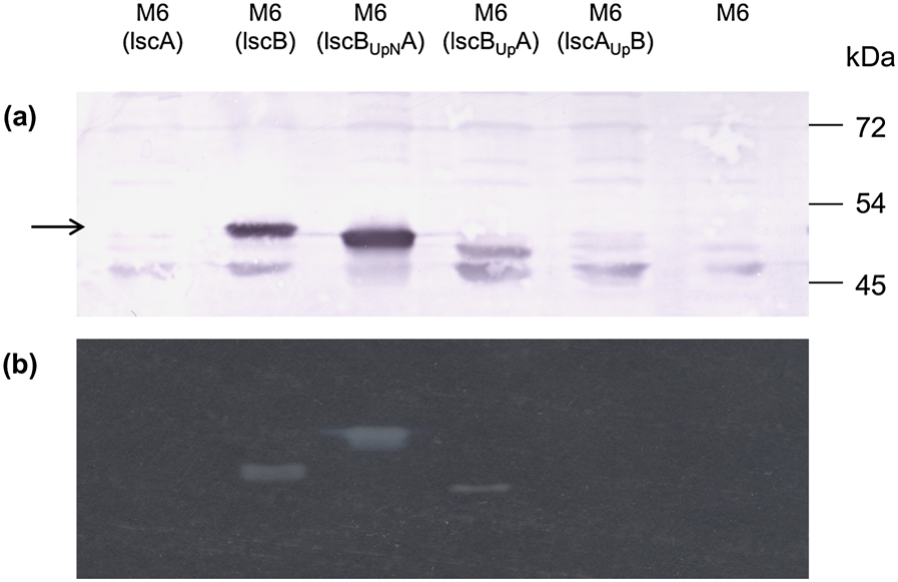
**Detection of levansucrase. (a)** Western blot analysis: 10 μg of total proteins were separated by 10% SDS-PAGE, transferred onto PVDF membrane, hybridized with anti-Lsc antiserum and detected using BCIP/NBT. The dark bands (arrow) correspond to Lsc and the corresponding fusion proteins. **(b)** Zymogram: 100 μg of total proteins were separated by 10% native-PAGE and incubated in 5% sucrose solution overnight. The white bands indicate formation of levan after utilization of sucrose by Lsc and the fusion proteins.

To verify the molecular sizes of Lsc encoded by the individual fusion constructs, a Western blot analysis using Lsc-specific antibodies was performed (Figure 3a). Under denaturing conditions, it was interesting to observe that LscB_UpN_A migrated at an intermediate rate i.e. faster than LscB but slower than LscB_Up_A. The signal for LscB_Up_A was weaker than those representing LscB or LscB_UpN_A suggesting that the N-terminus of LscB might contribute to the expression level or stability of Lsc. In contrast, protein samples of PG4180.M6 transformed with LscA or LscA_Up_B did not show any signal specific for Lsc at all thus confirming that lack of levan formation was due to lack of the corresponding protein.

To check for the enzymatic function of Lscs encoded by the individual fusion constructs, zymographic detection was done with non-denatured total protein samples of transformed mutants (Figure 3b). The above reported levan forming ability of transformants M6(lscB), M6(lscB_UpN_A) and M6(lscB_Up_A) could be attributed to the enzymatic functioning of proteins or fusion proteins. As expected, native protein samples derived from M6(lscA) or M6(lscA_Up_B) did not exhibit any in-gel levan production (Figure 3b). An interesting observation was the altered electrophoretic mobility of the enzymatically active proteins. The LscB_UpN_A migrated slower as compared to LscB even though the predicted molecular masses of both proteins were almost identical (~47.6 kDa) suggesting possible differences in the respective protein charges. In accordance with the Western blot results, LscB_Up_A seemed to be less expressed than LscB or LscB_UpN_A suggesting an important role of the N-terminus for transcriptional or translational processes.

### MALDI-TOF analysis

**Figure 4:**
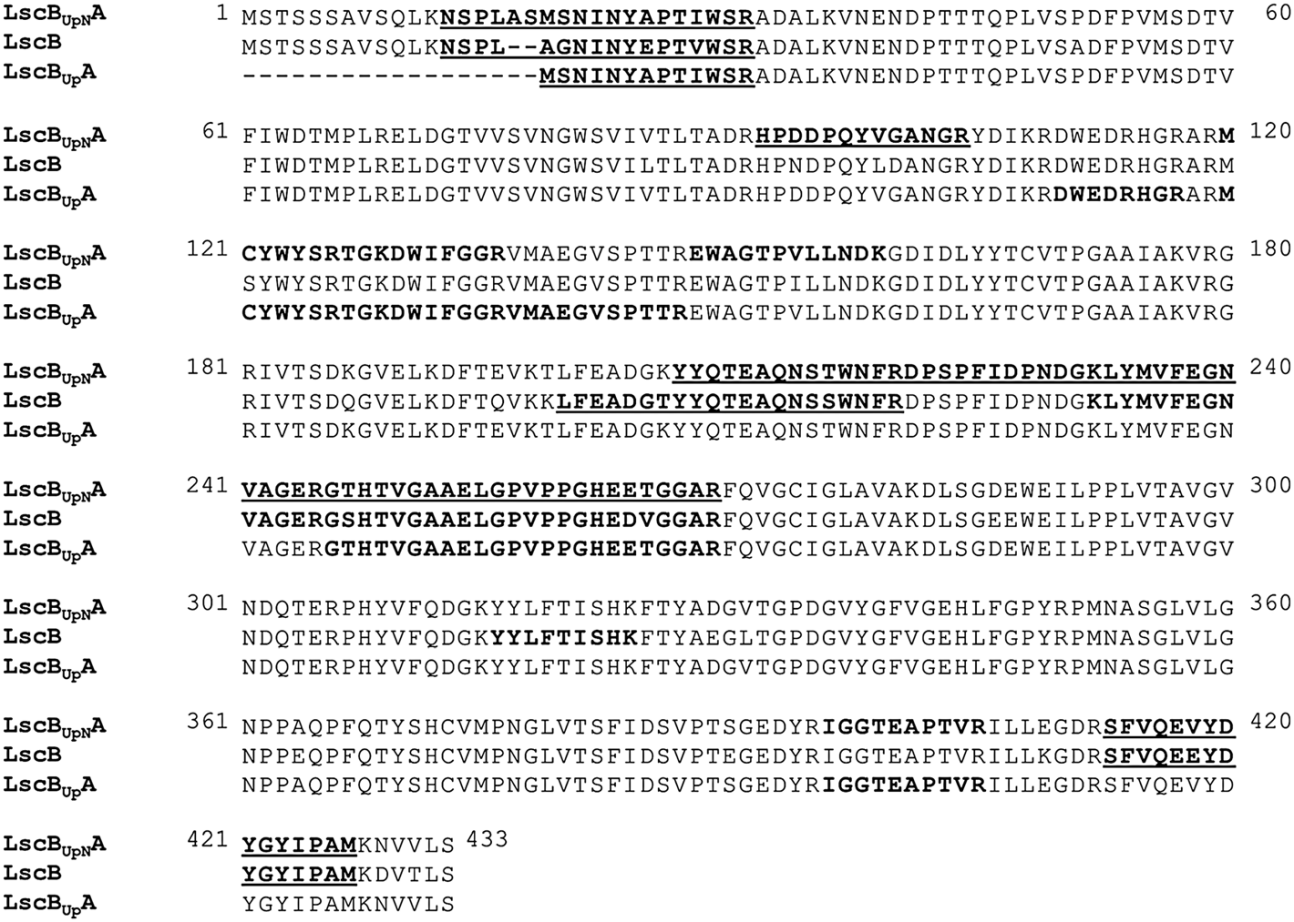
**Amino acid sequence alignment of LscB**_**UpN**_**A, LscB and LscB**_**Up**_**A.** Fragments in bold indicate peptides recovered from MALDI-TOF analysis. The underlined fragments indicate recovered peptides which are unique to that protein.

The altered electrophoretic migration rate of LscB_UpN_A as compared to LscB during the native gel protein separation suggested that the two proteins were indeed different although their predicted protein sizes were almost identical. To demonstrate that LscB_UpN_A produced a unique and novel enzyme and to show that the other two transformants indeed also produced the intended Lsc proteins, we subjected the levan-forming fusion proteins to MALDI-TOF analysis. The peptides recovered in the MALDI-TOF analysis are shown in Figure 4. The recovered peptides gave rise to an overall good coverage in the protein sequences (Table 1). Some of the peptides recovered were unique to each protein (Figure 4, underlined). E.g., peptides SFVQEVYDYGYIPAM from LscB_UpN_A and SFVQEEYDYGYIPAM from LscB were located at the same position, namely 413–427, in the respective amino acid sequences of these proteins but had different masses, 1,782 Da as compared to 1,812 Da, indicating they were from different proteins. Similar differences were observed for the other peptide sequences shown in the Figure 4 indicating that the fusion constructs indeed led to the synthesis of novel fusion proteins or of the proteins intended despite the presence of similar upstream regions.

**Table 1:** Proteins identified by MALDI-TOF analysis

**NCBI accession number/gi**	**Protein description**	**Predicted molecular mass (Da)**	**Significant hit**	**MASCOT score**	**Peptides matched**	**Sequence coverage (%)**
13936820	LscB	47,603	LscB	101	10	31
3914944	LscB_UpN_A	47,621	LscA	110	12	33
416026576	LscB_Up_A	45,844	LscA	110	8	19

### Analysis of *lscA* fusion protein expression by qRT-PCR

**Figure 5:**
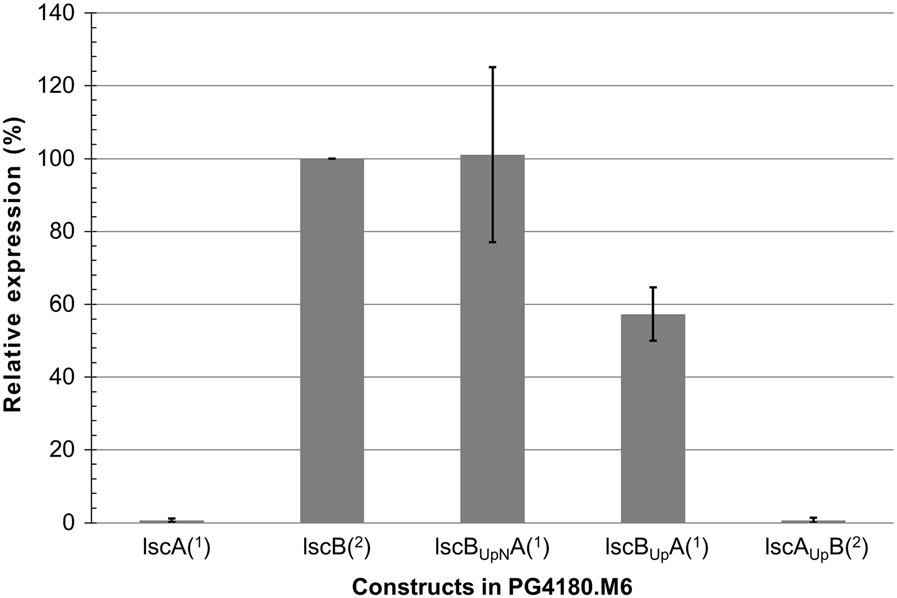
**Quantitative expression of different *lsc *genes and constructs in dependence of *lscB*.***lscB*_*UpN*_*A* shows similar levels of expression as *lscB* while *lscB*_*Up*_*A*, which does not contain the first 48 bp of *lscB* ORF, has lower expression. *lscA* and *lscA*_*Up*_*B* were not seen to be expressed. *lscA*, *lscB*_*UpN*_*A* and *lscB*_*Up*_*A* were detected using *lscA* primers (^1^) while the rest using *lscB* primers (^2^). The data represent the mean relative expression of 3 replicates ± standard deviations. Data were normalized to the highest expression value of *lscB*, which was set to 100%.

The difference in the amount of levan produced by LscB_Up_A as compared to LscB_UpN_A and LscB in the zymogram prompted us to check if this correlated at the RNA level. Samples were grown in HSC medium at 18°C and harvested at OD_600_ of 0.5 since *lsc* transcription is maximum at this optical density [23]. The total RNA was extracted from the cells and the expression of *lscB* and *lscA*_*Up*_*B* was checked by *lscB*-specific primers while that of *lscA*, *lscB*_*UpN*_*A* and *lscB*_*Up*_*A* was checked by *lscA*-specific primers. The results showed that, considering the standard deviation obtained for the samples, the *lscB*_*UpN*_*A* had expression levels similar to *lscB* (Figure 5) further supporting the results of the Western blot and zymogram. On the other hand *lscB*_*Up*_*A* had only 60% expression as compared to *lscB*. As was the trend seen in the Western blot and zymogram, *lscA* and *lscA*_*Up*_*B* had no expression. This indicated that even though the upstream region of *lscB* is sufficient to promote the expression of *lsc*, the expression level is enhanced by the presence 48-bp N-terminus of *lscB*.

### Analysis of native gene expression of *lscA* in *P. syringae* pathovars

**Figure 6:**
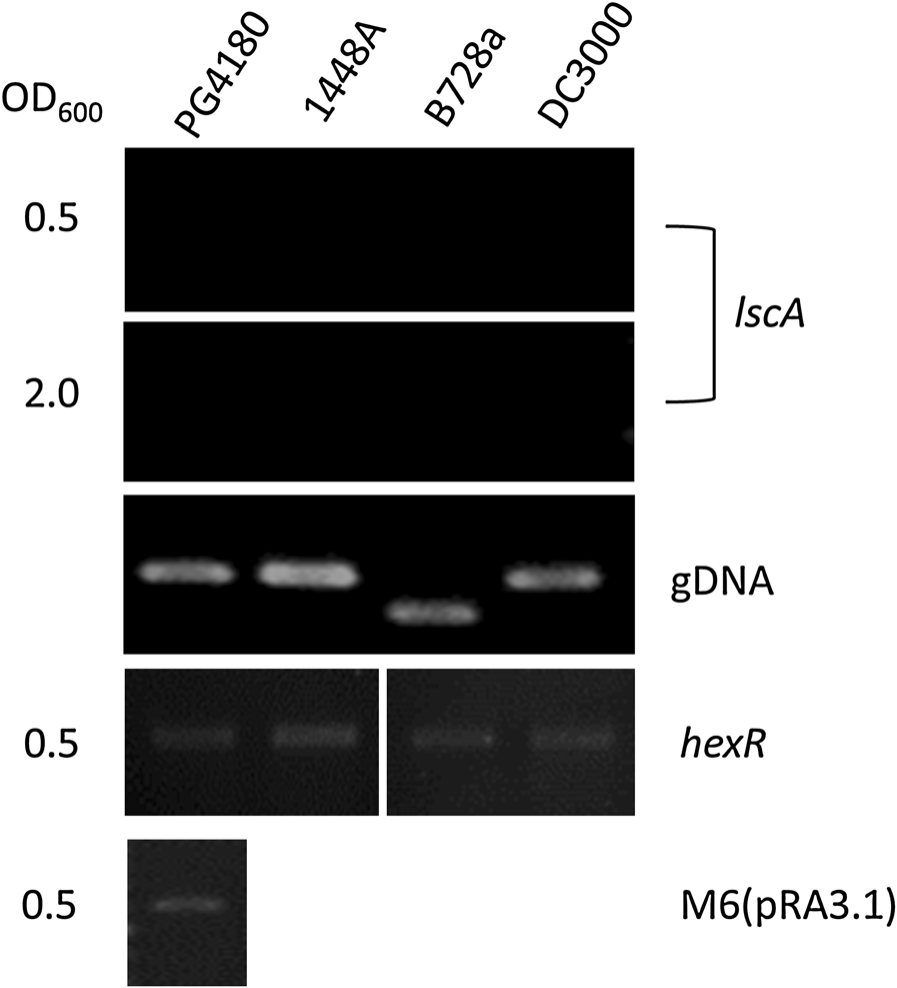
**Expression of *lscA *in different *P. syringae *pathovars.** The bacterial cells were harvested at OD_600_ of 0.5 and 2.0. Total RNA was extracted as described in the Materials and Methods followed by generation of cDNA. PCR amplification of *l*scA fragment on the total cDNA using strain-specific primers showed no amplicon (*lscA* panel) indicating no expression of *lscA*. Quality of the primers was checked by performing PCR amplification using genomic DNA (gDNA) as template. Amplification using an unrelated gene *hexR* (*hexR*) and artificially expressed *lscA* by P_*lac*_ [M6(pRA3.1)] signified correct reverse transcription.

Lack of expression of *lscA* had been shown before in *P. syringae* pv. glycinea PG4180 [10]. However, this has not been experimentally proven for other *P. syringae* pathovars. Consequently, possible expression patterns of *lscA* variants were also analyzed in the three *P. syringae* pathovars pv. phaseolicola 1448A, pv. syringae B728a and pv. tomato DC3000 using cDNA synthesis and PCR. No amplicon was detected in any of the four strains as shown in Figure 6 indicating that none of the *lscA* variants are expressed. The specificity of the primers was demonstrated by amplifying the *lscA* genes from corresponding genomic DNA, all of which gave amplicons of the expected sizes. The accuracy of reverse transcription was checked by amplifying a cDNA of a PG4180.M6 transformant carrying a recombinant *lscA* gene under the control of P_lac_, where *lscA* is known to be expressed [10]. Successful cDNA synthesis of total mRNA was also demonstrated by PCR amplifying the cDNA derived from the mRNA of the *hexR* gene, a hexose metabolism regulator [25]. Gene *hexR* gave an amplicon of expected size (Figure 6) indicating correct cDNA synthesis.

## Discussion

Genomic co-existence of three highly conserved genes coding for levansucrase is a feature unique to the plant pathogen *P. syringae* despite the fact that numerous other bacterial species harbor just a single copy of this gene in their genomes. Artificial expression of *lscA* from *P. syringae* under the control of the P_lac_ had been shown previously [10]. The same study also showed that *lscA* could not be expressed under its own promoter. Major differences between *lscA* and the natively expressed genes *lscB* and *lscC* are not found in the coding sequences but in their upstream DNA regions. The upstream regions of *lscB* and *lscC* represent a possible PAPE [24]. We previously hypothesized that this PAPE might harbor regulatory sites required for expression of levansucrase and general sugar metabolism in *P. syringae*. Herein, the PAPE of *lscB* was fused to the coding sequence of *lscA* and thus proven for its transcriptional activity in *P. syringae*.

The nucleotide sequence of the predicted PAPE consists of two parts, the upstream region of *lscB* and the first 48-bp coding for the N-terminus of LscB. The importance of these 48-bp of the ORF for the expression was tested by generating fusion constructs of the upstream region and *lscA* with or without these coding nucleotides. Transformants carrying either of the two fusion constructs produced levan similar to the PG4180.M6 mutant complemented with *lscB*. Western blotting, zymographic detection, and qRT-PCR analyses confirmed these results but also allowed a more detailed view; native *lscB* and the *lscB*_*UpN*_*A* fusion had similar mRNA expression levels while that of the fusion *lscB*_*Up*_*A*, which lacked the 48-bp of N-terminal LscB-coding region, had less. Consequently, one might speculate that although the -450 bp upstream DNA region of *lscB*, which includes the TSS as determined in this study, is sufficient for expression of *lscA*, the first 48-bp of the *lscB* ORF increase the level of its expression. Since our respective results of Western blotting and zymographic detection of Lsc activity were indistinguishable from each other, it could be concluded that the N-terminus of LscB might not be involved in altering of enzymatic activities.

A peculiar observation was the electrophoretic migration of the individual proteins or fusion proteins in polyacrylamide gels. The observed faster migration of LscB_UpN_A as compared to LscB under denaturing conditions could potentially be attributed to the apparent mass shift for two proteins with nearly identical molecular masses as described earlier [26]. Interestingly, the migration of LscB_UpN_A was significantly slower than that of LscB under native conditions. This finding might demonstrate that modest changes in the protein’s surface charge might result in significant alterations of electrophoretic mobility [22,27,28].

Although the different migration rates of the proteins or fusion proteins under native or denaturing conditions suggested that the synthesized proteins were indeed different from each other, a MALDI-TOF analysis of each of the proteins was conducted using protein samples from zymograms. The produced levan surrounding the proteins did not seem to impact mass spectrometric analysis. The MASCOT score for each of the identified proteins was above the significance threshold of 100. The sample from the PG4180.M6(lscB) sample gave LscB from *P. syringae* pv. phaseolicola 1448A as the first significant match which was in line with the high homology of the respective genes in the close relatives pv. glycinea and pv. phaseolicola [24]. The sample from PG4180.M6(lscB_Up_A) which should synthesize only LscA gave the first significant match as LscA from *P. syringae* pv. glycinea race 4 strain. This proved that the *lscB*_*Up*_*A* fusion actually synthesized an active LscA and confirmed earlier findings that artificial expression of LscA of PG4180 leads to levan formation [10]. Although the majority of obtained peptides for the sample representing LscB_UpN_A were LscA-borne as expected, the unique N-terminal 2,122-Da peptide NSPLASMSNINYAPTIWSR could be detected. This peptide is a consequence of the presence of the *Nhe*I restriction site coding for the amino acid residues alanine and serine. Oxidation of methionine, which was chosen as a variable modification parameter, added another 16 Da to the peptide mass which subsequently increased the mass of the NSPLASMSNINYAPTIWSR fragment to 2,138 Da. This mass was exactly the same as the mass of a recovered peptide which did not find a match during the NCBI search since the respective fusion peptide is not present in the database. Thus, the synthesis of the LscB_UpN_A fusion protein could also be proven.

The majority of previous LscA-related studies have been performed with *P. syringae* pv. glycinea PG4180 [9,10,23,24]. However, thus far, there was no evidence for a lack of *lscA* expression in other pathovars of *P. syringae*. Since the genomes of *P. syringae* pv. phaseolicola 1448A, pv. syringae B728a and pv. tomato DC3000 are fully sequenced [19-21], template-specific oligonucleotide primers for cDNA-based mRNA detection could be designed. Although mRNA samples were extracted during different growth stages, namely, early-logarithmic and late-logarithmic phase, no amplicons could be detected in any of the strains suggesting that *lscA* variants were not expressed. PCR amplification, using respective genomic DNA as template, proved that the primers were binding correctly. An independent gene, *hexR*, coding for a conserved hexose metabolism regulator protein HexR, was chosen to see if the total mRNA had been reverse transcribed correctly [25]. This PCR amplification gave correct sized amplicon of 880-bp for all the four strains demonstrating the accuracy of the used method. PCR amplification was also performed on the cDNA obtained from mRNA samples of PG4180.M6 containing *lscA* under the control of P_*lac*_. This experiment gave the same-sized amplicon as for genomic DNA again proving the accuracy of the method.

In summary, we propose that *lscA* could be an ancestral Lsc variant in *P. syringae* as suggested by Srivastava *et al*. [24]. During evolution, the inactive promoter perhaps did not allow expression of *lscA* after this gene had potentially been introduced to an ancestral *P. syringae*. An evolutionary gene duplication of *lscA* followed by an insertion of a prophage-borne PAPE might have led to a new *lsc* variant, i.e. *lscB* which in turn got duplicated yielding *lscC* or vice-versa. As a result of this evolutionary process, two functional and expressed *lsc* genes emerged in the plant pathogen, for which utilization of sucrose, and perhaps levan formation, might be particularly important. The advantage of an additional *in planta* fitness-increasing and possibly virulence-promoting factor [29] could have helped this organism to selectively establish itself as a potent plant pathogen. As a consequence of this hypothesis, one could speculate on a loss of the supposedly non-expressed *lscA* during further evolutionary steps, a phenomenon also previously hypothesized by Smits *et al*. [30].

## Conclusions

The differential expression of levansucrases in *P. syringae* was long known, but not tested. In this study, we have potentially solved the previously unexplainable phenomenon that *P. syringae* is the only organism possessing multiple levansucrase-encoding genes. We demonstrated the importance of the upstream region as well as the N-terminus of *lscB/C* required for the expression of Lsc in *P. syringae*. The upstream region of *lscA* does not seem to promote *lsc* expression. With careful controls, herein we also demonstrated that *lscA* is not expressed in other *P. syringae* pathovars.

## Methods

### Bacterial strains, plasmids and growth conditions

Bacterial strains, plasmids and oligonucleotides used in this study are listed in Tables 2 and 3. *E. coli* DH5α was used as the cloning host [31] and grown in Lysogeny Broth (LB) medium at 37°C. *P. syringae* cultures were grown in HSC medium (0.8 mM MgSO_4_.7H_2_O, 30 mM KH_2_PO_4_, 16 mM K_2_HPO_4_, 2 mM KNO_3_, 20 μM FeCl_3_, 19 mM NH_4_Cl, 100 mM glucose) [32] at 18°C. Bacterial growth in liquid media was monitored by measuring the optical density at 600 nm (OD_600_) and harvested for (i) protein sampling at an OD_600_ of 2.0 or (ii) RNA extraction and cDNA synthesis at an OD_600_ of 0.5 and 2.0. Antibiotics were added to the media at the following concentrations (μg ml^-1^): ampicillin 50; tetracycline 25, and chloramphenicol 25.

**Table 2:** Bacterial strains and plasmids used in this study

**Strain**	**Description**	**Reference or source**
*Pseudomonas syringae*		
pv. glycinea PG4180	Wild type, levan+	R. Mitchell
pv. phaseolicola 1448A	Wild type, levan+	[33]
pv. syringae B728a	Wild type, levan+	[34]
pv. tomato DC3000	Wild type, levan+	D. Cuppels
*Pseudomonas syringae* pv. glycinea PG4180		
PG4180.M6	Sp^r^, Gm^r^, *lscB lscC* mutant of PG4180, levan-	[10]
PG4180.M6(pRA3.1)	Sp^r^, Gm^r^, Tc^r^, *lscB lscC* mutant of PG4180, containing *lscA* under control of P_*lac*_ on 3.1-kb *Pst*I fragment in pRK415	[10]
*Escherichia coli*		
DH5α	supE44 D*lac*U169 (F80 *lacZ*DM15) *hsdR*17 *recA*1 *endA*1 *gyrA*96 *thi*-1 *relA*1	[31]
Plasmids		
pRK2013	Km^r^, helper plasmid	[35]
pLB7.2	Ap^r^, contains *lscB* on 7.2-kb *Eco*RV insert	[10]
pBBR1MCS	Cm^r^, broad-host-range cloning vector	[36]
pBBR1MCS-3	Tc^r^, broad-host-range cloning vector	[36]
pBBR3-500-lscB	Tc^r^, *lscB* gene with −500-bp upstream sequence in pBBR1MCS-3	[24]
pBBR3(lscA)	Tc^r^, *lscA* gene containing insert from pRA3.1 in PBBR1MCS-3 not under control of P_*lac*_	This study
pBBR3(lscB_UpN_A)	Tc^r^, fusion of 518-bp upstream region of *lscB* (including first 48-bp of coding region) and *lscA* (including start codon and downstream region) in pBBR1MCS-3	This study
pBBR3(lscB_Up_A)	Tc^r^, fusion construct of 470-bp upstream region of *lscB* (without N-terminus) and *lscA* (including start codon and downstream in pBBR1MCS-3	This study
pBBR3(lscA_Up_B)	Tc^r^, fusion of 550-bp upstream region of *lscA* and *lscB* (including start codon and downstream region) in pBBR1MCS-3	This study

Ap, Ampicillin; Cm, Chloramphenicol; Gm, Gentamycin; Km, Kanamycin; Sp, Spectinomycin; Tc, Tetracycline; ^r^, resistant.

**Table 3:** Oligonucleotide primers used in this study

**Oligonucleotides**	**Nucleotide sequence (5’ - 3’)**^**†**^
pe.BC.PG ~ 150 bp	GTCACCCATGCGGGCCAGCAG
lscB_UpN_f	CCC*AAGCTT*CGATTGCAAGCTGATACACGTACC
lscB_UpN_r	TAG*GCTAGC*TAGAGGACTATTTTTGAG
lscA_ORF_f	CTA*GCTAGC*ATGAGTAACATCAATTAC
lscA_ORF_r	CCC*AAGCTT*CGGACGTCATCCTGATCGACAC
lscB_Up_r	TAG*GCTAGC*AATTGATACCTTTAAATAGCTTTGGGAG
lscA_Up_f	CG*GGATCC*AGCAAAGCGCTGTAAAACAGG
lscA_Up_r	CTACTA*GCTAGC*GATGATGTCCTTTATTGGCGC
lscB_ORF_f	GC*TCTAGA*TGTCCACTAGCAGCTCTGCTGTAA
lscB_ORF_r	CCC*AAGCTT*TCAGTATTACGGATACGATGAGC
lscA_gly_f	TAAGCCCGGATTTTCCGGTC
lscA_gly_r	TACTGTATGCGTGCCGCGTT
lscA_pha_f	TCACGCTGACGGCTGACCGC
lscA_pha_r	GCCTACTGTATGCGTGCCGCG
lscA_syr_f	TCACGCTGACAGCTGATCGC
lscA_syr_r	ACCAACGGTATGCGTACCGC
lscA_tom_f	ATCACCCTGACAGCCGACCG
lscA_tom_r	ACCGACAGTATGTGAACCCCGCT
lscA_f_RT	ATGAGTAACATCAATTACGCACCC
lscA_r_RT	TACTTTGGCAATTGCCGCAC
lscB_f_RT	CTCTGCTGTAAGCCAGCTCAA
lscB_r_RT	CGGGTGTGACGCAGGTGTAA
gyrA_fw	CGAAGAGCTGGAAGTGATCC
gryA_rv	GACGCTGAGCCTGATAGACC
hexR_fw	ATGGACCGCGTAAGAAAC
hexR_rv	TCAGCCTTGATCCTCGATCGG

^†^Restriction sites in the primers are in italics: GAGCTC – *Sac*I, AAGCTT - *Hin*dIII, GCTAGC - *Nhe*I, GGATCC - *Bam*HI, TCTAGA – *Xba*I.

### Molecular genetic techniques

Plasmid isolation, restriction enzyme digests, agarose and polyacrylamide gel electrophoreses, electroporation, PCR, and other routine molecular methods were performed using standard protocols [31]. Nested deletion analysis of the upstream region of *lscB* in plasmid pRB7.2 [10] was conducted using the Erase-a-Base kit (Promega, Madison, USA). For analysis of the *lsc* upstream regions, PCR was used to generate products covering the respective regions (Table 3). PCR products of the *lsc* upstream regions were cloned in vectors pBBR1MCS or pBBR1MCS-3 [36].

### Determination of transcriptional start site

Bacteria were incubated in HSC medium at 18°C to an OD_600_ of 0.5 and harvested by mixing 15 ml of the culture with an equal volume of chilled killing buffer (20 mM Tris–HCl [pH7.5], 20 mM NaN_3_). This mixture was centrifuged at 4°C for 15 min at 3,220 × g. Total RNA was isolated from the cell pellets by acid phenol/chloroform extraction as described previously [37]. For primer extension analysis, 4 pmol of ^32^P-labeled primer pe.BC.PG ~ 150 bp (Table 3) were annealed with 10 μg of total RNA and reverse transcription was performed with M-MLV Reverse Transcriptase (Invitrogen, Karlsruhe, Germany). Nucleotide sequencing using 5 μg of plasmid pLB7.2 (Table 2) and primer pe.BC.PG ~ 150 bp was done with the Sequenase Version 2.0 DNA Sequencing Kit (USB, Cleveland, USA) according to the manufacturer’s recommendation. The extension product and sequencing reaction were resolved on a 6% polyacrylamide sequencing gel. Signal detection was performed using a FLA-3000 phosphorimager (Raytest, Straubenhardt, Germany) according to the manufacturer’s recommendations.

### Generation of fusion constructs

All genes or DNA fragments were obtained by PCR amplification unless otherwise stated. All restriction enzymes used were obtained from Thermo Fisher Scientific Biosciences (St. Leon Rot, Germany). The nucleotide sequencing was done by Eurofins MWG Operon (Ebersberg, Germany).

Generation of *lscB*_*UpN*_*A* and *lscB*_*Up*_*A*: The sequences of the 518-bp PAPE and the 470-bp *lscB* upstream region without the 48-bp coding sequence, respectively, were ligated to the N-terminus of the 1,748-bp *lscA* fragment using T4 DNA Ligase (Thermo Fisher Scientific Biosciences) after treating the DNA with restriction enzyme *Nhe*I. The ligation products were then treated with *Hin*dIII, analysed by agarose gel electrophoresis, and the bands corresponding to the fusion products (2,284 and 2,224 bp, respectively) were purified from the gel using GeneJET Gel Extraction kit (Thermo Fisher Scientific Biosciences). The purified fusion products were ligated into pBluescript-KS(II) using *Hin*dIII in such a way that the fusion products were under control of the vector-borne *lac* promoter (P_*lac*_). Formation of levan on LB agar containing 5% sucrose indicated a functional *lscA* gene driven by the P_*lac*_. The PAPE and *lscB* upstream regions were sequenced to exclude any possibility of mutations. The fusion products were then cloned into the broad host-range vector pBBR1MCS using *Hin*dII in order to ligate them in opposite orientation to the P_*lac*_ and then cloned into pBBR1MCS-3 using restriction enzymes *Pst*I and *Xho*I to keep the same opposite orientation with respect to P_*lac*_ as in case of pBBR1MCS. The constructs were introduced into mutant PG4180.M6 via electroporation.

Generation of *lscA*_*Up*_*B*: A similar cloning strategy was used to generate the *lscA*_*Up*_*B* construct. The C-terminus of the 550-bp PCR-amplified *lscA* upstream region and the N-terminus of the 1,704-bp PCR-amplified ORF *lscB* were ligated using a combination of restriction enzymes *Xba*I and *Nhe*I which generate compatible DNA ends. This ligation product was treated with endonucleases *Bam*HI and *Hin*dIII and subsequently ligated into pBluescript-SK(−). The constructs were cloned into pBBR1MCS using restriction enzymes *Bam*HI and *Hin*dII in order to ligate them in opposite orientation to the P_*lac*_ and then into pBBR1MCS-3 using restriction enzymes using *Xba*I and *Apa*I to keep the same opposite orientation with respect to P_*lac*_ as in case of pBBR1MCS.

### Immunological and enzymatic detection of Lsc

Total proteins from PG4180.M6 and PG4180.M6 transformants harboring the *lsc* fusion constructs were obtained as described previously [23]. For immunological detection of the Lsc enzyme, total proteins were separated by 10% SDS-PAGE and Western blot experiments were performed with total protein fractions using polyclonal antibodies raised against purified Lsc as reported earlier [10]. Zymographic detection of Lsc was done as described previously by separating the total proteins by 10% native-PAGE and incubating the gels in 5% sucrose solution [10]. Bacterial cells grown on mannitol-glutamate agar plates with 1.5% agar and 5% sucrose were used for the qualitative visualization of Lsc activity, which led to levan formation in form of a mucoid, dome-shaped colony morphology. Lsc activity was quantified by measuring the amount of glucose liberated during incubation with sucrose using the Gluco-quant Glucose/HK assay kit (Roche Diagnostics, Mannheim, Germany) at an absorbance of 340 nm. One unit of Lsc activity corresponded to the amount of enzyme which liberates 1 μmol glucose per minute from sucrose. The experiments were repeated three-fold and mean values were expressed as the quantity of glucose release.

### MALDI-TOF mass spectrometric analysis

Total proteins were separated using 10% native-PAGE and incubated in 5% sucrose solution overnight [10]. As soon as in-gel levan formation became apparent, the corresponding bands were cut out from the gel and subjected to an in-gel proteolytic cleavage using modified porcine trypsin (Promega, Madison, WI) as adapted from previous reports [38-40]. Trypsin digestion was carried out for 12–16 h at 37°C, and peptide samples were directly used for MALDI-TOF MS exposure using an Autoflex II TOF/TOF mass spectrometer (Bruker Daltonics, Bremen, Germany) equipped with a 337 nm nitrogen laser and operated with FlexControl 3.0 software. The matrix used was 1 mg ml^−1^ of a-cyano-4-hydroxycinnamic acid (HCCA; Bruker Daltonics) disolved in acetone and mixed with two volumes of ethanol. Peptide samples were acidified with 0.5% TFA in a ratio of 1:1 (v/v) and mixed with the HCCA solution in a ratio of 1:1 (v/v). Samples of 0.5 μL were spotted and air-dried on MTB AnchorChip targets with an anchor diameter of 600 μm (Bruker Daltonics). Spots were twice rinsed with 2 μL of 10 mM monobasic ammonium phosphate solution for ~5 s, dried, and exposed to MALDI-TOF MS in positive-ion reflection mode with the laser offset set to 67% +/− 15% and an acquisition range of 800–4,000 Da. A signal-to-noise ratio of 6 was applied for peak identification using the Mascot search engine [41] from Biotools software 3.1. Mass lists were compared with NCBI databases and the Mascot score probability set for p <0.05. Peptide sequence analyses was done using the ExPASy bioinformatics resource portal [42].

### Analysis of *lsc* gene expression by quantitative Reverse Transcriptase polymerase chain reaction (qRT-PCR)

Total RNA was isolated by acid phenol/chloroform extraction as described previously [11]. The yield and the purity of RNA were determined by measuring absorption at 260 nm. Total mRNA samples were treated with TURBO DNA-free (Applied Biosystems, Darmstadt, Germany) to remove remaining traces of genomic DNA as described by the manufacturer’s recommendation. SYBR-green based qRT-PCR was performed with 5 ng RNA template and 100 μM primer with QuantiTect SYBR Green one-step RT-PCR Kit (Qiagen, Hilden, Germany) according to the manufacturer’s instructions. The thermocycler program comprised an initial step of 95°C for 15 min followed by 40 cycles of 95°C for 30 s, 58°C for 30 s, 72°C for 30 s. Reactions were performed with biological triplicates in a Mastercycler ep realplex2 real-time PCR system (Eppendorf, Hamburg, Germany) as described by the manufacturer using their universal program. Reactions with no addition of reverse transcriptase served as negative control and proved the absence of DNA contamination. Specificity of amplification was assessed by analyzing the melting curve of the amplification product. Primers to amplify *lscB* were used for constructs *lscB* and *lscA*_*Up*_*B* while primers to amplify *lscA* were used for constructs *lscA*, *lscB*_*UpN*_*A* and *lscB*_*Up*_*A.* All the results were normalized to amplification of the cDNA of *gyrA* (PSPPH3667) as described previously [43].

### Analysis of *lscA* gene expression by Reverse-Transcriptase polymerase chain reaction (RT-PCR)

Template-specific primers were designed for the respective *lscA* variants of *P. syringae* pv. glycinea PG4180, pv. phaseolicola 1448A, pv. syringae B728a, and pv. tomato DC3000. Bacterial cells were grown in HSC medium and harvested at an OD_600_ of 0.5 as well as 2.0. RNA was extracted by acid phenol/chloroform extraction method [11]. An RT-PCR was performed on total mRNA using RevertAid First Strand cDNA Synthesis Kit (Fermentas) as recommended by the manufacturer. The strain-specific *lscA* primers were used to check for presence of an *lscA* mRNA by PCR using cDNA as template. Regular PCR with the same primer-pairs and genomic DNA as template were used as controls. The thermocycler program was as follows: 1 cycle of 95°C for 90 s; 25 cycles of 95°C for 15 s, 66°C for 15 s, 72°C for 30 s; 1 cycle of 72°C for 5 min. The results were analyzed by 1% agarose gel electrophoresis.

### Bioinformatics analyses

Vector NTI Advance 10.1.1 (Life Technologies, California, USA) was used for the nucleotide, amino acid sequence alignments, as well as for generating genetic maps. BLAST-N and BLAST-P programs were used for online sequence analyses [44]. The website http://www.pseudomonas.com was consulted for the determination of *P. syringae* gene orthologs and paralogs [45].

## Abbreviations

Lsc: Levansucrase; MALDI-TOF: Matrix-assisted laser desorption/ionization-time of flight; PAPE: Phage-associated promoter element; PG4180: *Pseudomonas syringae* pv. glycinea PG4180.

## Competing interests

All authors of the study (SK, ASr, DP, ASt and MU) declare that there are no competing interests (whether political, personal, religious, ideological, academic, intellectual or commercial) or any other activities influencing the work.

## Authors’ contributions

SK generated the fusion constructs, performed the levan formation, Western blot, zymogram, RT-PCR and qRT-PCR assays; ASr determined the transcriptional start site; DP generated and analysed a fusion construct; ASt conducted the MALDI-TOF data acquisition and analysis; MU coordinated the study; SK and MU prepared and revised the manuscript draft. All authors contributed to the preparation and approval of the final manuscript.

## Authors’ information

SK – Department of Molecular Microbiology, Molecular Life Sciences Research Center, Jacobs University Bremen, Germany; ASr - Current Address: Department of Experimental Limnology, Leibniz-Institute of Freshwater Ecology and Inland Fisheries, Stechlin, Germany; DP – Department of Biochemical Engineering, Molecular Life Sciences Research Center, Jacobs University Bremen, Germany; ASt – Department of Molecular Microbiology, Molecular Life Sciences Research Center, Jacobs University Bremen, Germany; MU – Department of Molecular Microbiology, Molecular Life Sciences Research Center, Jacobs University Bremen, Germany.
